# Forgiveness, Representative Judgement and Love of the World: Exploring the Political Significance of Forgiveness in the Context of Transitional Justice and Reconciliation Debates

**DOI:** 10.1007/s11406-016-9726-7

**Published:** 2016-06-08

**Authors:** Maša Mrovlje

**Affiliations:** Politics and International Relations, University of Edinburgh, Edinburgh, UK

**Keywords:** Political forgiveness, Representative judgement, Tragedy of political action, Reconciliation with reality, Love of the world

## Abstract

The article examines the political challenge and significance of forgiveness as an indispensable response to the inherently imperfect and tragic nature of political life through the lens of the existential, narrative-inspired judging sensibility. While the political significance of forgiveness has been broadly recognized in transitional justice and reconciliation contexts, the question of its importance and appropriateness in the wake of grave injustice and suffering has commonly been approached through constructing a self-centred, rule-based framework, defining forgiveness in terms of a moral duty or virtue. Reliant on a set of prefabricated moral standards, however, this approach risks abstracting from the historical, situated condition of human political existence and thus arguably stands at a remove from the very quandaries and imperfections of the political world, which it purports to address. Against this background, this article draws on Albert Camus’s and Hannah Arendt’s aesthetic, worldly judging sensibility and its ability to kindle the process of coming to terms with the absurd, and perhaps unforgivable character of reality after evil. As an aptitude to engage the world in its particularity, plurality and contingency rather than seeking to subdue and tame it under prefabricated standards of thought, namely, worldly judgement is able to reveal how past tragedies have arisen from the ambiguity of human engagement in the world and thereby also elicit the distinctly human capacities of beginning anew and resisting such actions in the future. As such, I suggest, it is well-suited to bring into clearer focus and confront the main *political* challenge and significance of forgiveness: how to acknowledge the seriousness of the wrongs committed, yet also enable the possibility of a new beginning and restore among former enemies the sense of responsibility for the shared world.

## Introduction

While not commonly examined in its potential political capacity in the field of philosophical literature, forgiveness has been broadly praised and called for for its moral and political significance in transitional justice and reconciliation contexts. It is considered to be a highly valuable response to wrongdoing able to break the vicious cycles of resentment and vengeful violence, renew and reform broken relationships, and pave the way for the society to move on into a democratic future (see e.g. Minow 1998, 14). At the same time, the insistence on the importance of forgiveness remains mired in a paradoxical recognition that the decision to forgive mass injustice and suffering – what would seem to be “unforgivable” – may be not only impossible but also morally undesirable, enabling further evasions of responsibility and ultimately betraying the sense of justice. This paradox, in turn, often is approached through constructing a normative, self-centred, rule-based framework, which, defining forgiveness in terms of a moral duty or virtue, is evoked to resolve the question of whether and when past evils and wrongs should be forgiven (see Minow 1998; Wiesenthal 1998; Gobodo-Madikizela 2004; see also MacLachlan 2009, 135–9; Pettigrove 2006, 490). Reliant on a set of prefabricated moral standards, however, this approach risks abstracting from the historical, situated condition of human political existence and thus arguably stands at a remove from the very quandaries and imperfections of the political world, which it purports to address. Against this background, this article draws on the existential, narrative-inspired and representative judging sensibility oriented by the principle of love of the world to bring to the forefront the distinctively political relevance of forgiveness. Specifically as developed in the work of Albert Camus and Hannah Arendt, aesthetic, worldly judgement is characterized as an aptitude to engage the world in its particularity, plurality and contingency rather than seeking to subdue and tame it under prefabricated standards of thought. As an ability to thereby reveal how past tragedies have arisen from the ambiguity of human engagement in the world and constantly disclose the human character of political affairs, worldly judgement arguably stands at the core of the difficult process of coming to terms with the absurd, and perhaps unforgivable character of reality after evil and of eliciting the distinctly human capacities of resisting such actions in the future. As such, I suggest, it is well-suited to bring into clearer focus and confront the main *political* challenge and significance of forgiveness as an indispensable response to the inherently imperfect and tragic nature of political life: how to acknowledge the seriousness of the wrongs committed, yet also enable the possibility of a new beginning.^1^ Specifically, I argue, it is able to foreground judgement on the appropriateness of forgiveness as an activity underlain by the situated and ambiguous process of assuming responsibility for the common world.

The argument proceeds as follows. In the first section, I explore Arendt’s insights into the political significance and challenge of forgiveness as it arises from the tragic nature of political action in the world and point to the inadequacy of the predominant moral discourse on forgiveness in adequately recognizing and confronting it. In turn, the section discloses the distinct political relevance of the aesthetic, representative judging sensibility in its ability to inspire the process of reconciliation with reality, with the often absurd and always plural character of the world. The second section illuminates the suggested shift of focus from a moral to a worldly perspective on forgiveness through two chosen literary works, Pumla Gobodo-Madikizela’s *A Human Being Died That Night* and Vitomil Zupan’s *Minuet for Guitar.* In the third section, on this basis, I elaborate on how narrative-inspired, worldly judgement and its underlying purpose of reconciliation with reality can illuminate forgiveness in its political significance by foregrounding it as a situated practice undertaken for the sake of human plurality and the common world.

## The Political Importance and Paradox of Forgiveness, and the Limitations of the Predominant Moral Discourse

Accompanying the proclamations on the worldly importance and value of forgiveness in transitional contexts, is the equally strongly voiced recognition of its ambivalent nature. On the one hand, a judgement to grant forgiveness is said to empower the victims of atrocity. Through relinquishing the often disempowering and self-destructive feelings of resentment and hatred and forgiving the perpetrators, the victims can reclaim their dignity. While affirming the suffering endured and the moral wrongfulness of the actions done to them, the argument goes, they can thus also renew their relationship with former oppressors and contribute to the broader project of reconciliation and societal reconstruction after violent and divided pasts (see e.g. Minow 1998, 14–15). On the other hand, however, forgiveness might also entail a further humiliation and devaluation of the victim, testifying to his or her inability to view and affirm the past suffering as an unjust and unacceptable violation that should instead rightly invite outrage and indignation (Minow 1998, 17–18). This danger is especially pronounced in cases when forgiveness is granted on behalf of the victims by governments or public officials in the form of, for instance, amnesty or impunity. As such, as it has often been emphasized, forgiveness may amount to nothing less than a betrayal of the victims’ suffering, involving as it may do not only a foregoing of just punishment but also a refusal to even acknowledge the harm done (Minow 1998, 16–17).

This difficulty can be seen as underlain by a more fundamental dilemma, well-echoed in Arendt’s explicit recognition of the importance (and paradox) of forgiveness in terms of the way it stems from the tragic nature of political action in the world. As opposed to vengeance as “the natural, automatic reaction to transgression” that stirs into motion “the relentless automatism of the action process,” forgiveness is distinguished as “the only reaction which does not merely re-act but acts anew and unexpectedly, unconditioned by the act which provoked it” (Arendt 1958, 241). Arendt then affirms forgiveness as a form of political action, capable of effecting a meaningful change in the political world. In this focus, she prefigures recent attempts to explore the human faculty of forgiveness not as a duty or virtue, but “a moral power” which has important performative dimensions and is not reducible to a change in emotional or mental states (see Card 2002, 173–188; see also e.g. MacLachlan 2009, 145). Forgiveness is an all-important political capacity, Arendt holds, in light of political action’s “irreversibility:” the fact that we always act into and in the midst of the world constituted by a plurality of human actors, wills and intentions and that the consequences of each of our actions are therefore bound to be “unending, infinite, and ultimately uncontrollable” (Arendt 1958, 236–7). Forgiveness is crucial because it “serves to undo the deeds of the past” in the sense of releasing us from the overwhelming consequences of our past actions that might stupefy our potentials to engage our freedom in the world in the future (Arendt 1958, 237). As conveyed most clearly in Arendt’s insight that “the deed is forgiven for the sake of who did it,” then, forgiveness is explicitly praised for its ability to kindle the human potentials for political action and an affirmation of a new beginning (Arendt 1958, 241). At the same time, however, Arendt’s take on forgiveness also carries a response to her radical awareness of the capacity of political action to bring about “incalculable evil.” Given political action’s unpredictability, its capacity to initiate a plethora of unintended consequences and processes that return to the agent in alien, essentially unrecognizable forms (Arendt 1958, 190–2), it can radically confound our ability of judging right from wrong, upset our deepest moral expectations and the established ways of relating to the world, and thus also challenge the very possibility of forgiveness. The main challenge of her time, as Arendt repeatedly pointed out, was the emergence of deeds human beings could neither punish nor forgive (Arendt 2003, 55).^2^

Yet, in the transitional justice literature, the prevalent emphasis on the political value of forgiveness as well as the accompanying predicament remain strongly underlain by a broader philosophical tendency to conceive of forgiveness as an essentially self-centred, inner, psychological or therapeutic exercise in overcoming resentment that enables personal healing and well-being after the traumatic experience of evil (see Pettigrove 2006, 488–9; MacLachlan 2009, 137, 145). The other-directedness of forgiveness, to be sure, is commonly recognized. When considering the appropriateness of forgiveness after grave wrongs, theorists have frequently evoked the need for sympathetic, narrative understanding and an appreciation of “moral luck,” that is, the fact that any action, its meaning and implications do not lie within our powers alone but are significantly conditioned by things beyond our control (Williams 1981).^3^ Scholars have argued that the practice of forgiving is closely linked to the capacity to place oneself into the situation of the other and look upon the world from his or her perspective, to weave the occurrences of wrongdoings and sufferings into stories, and thereby also effect “the temporalization of relations.” Narrative understanding, in this vein, can reveal how the past can be recognized as something that cannot be undone, ignored or avoided in a way that nevertheless affirms the human ability to respond to it and begin anew (Griswold 2007, xxii, 98, 100). An understanding of the context of the wrongdoing as well as the broader insight into the circumstances that have shaped the lives and choices of the perpetrator, for instance, are considered to be crucial in “*separating* the wrongdoer from the wrong which has been committed” (North 1998, 26). For thus it becomes possible to see in him or her a person that is more than whatever he or she has done, and also offer grounds for forgiveness based on the recognition that he or she can be a different person in the future (see e.g. North 1998, 24–6). Nonetheless, the understanding and judging processes commonly are conceived as empathetic exercises, in which the victim or the third person seeks to identify with the wrongdoer, to penetrate to the utmost kernel of his or her subjectivity and on this basis determine whether or not the person in question is deserving of forgiveness. Thence, indeed, arises the model form of forgiveness as an interpersonal, reciprocal moral relation (Griswold 2007, xvi). This sensibility, in other words, arguably remains mired in the self-centred focus, depicting judgement on whether or not to forgive as a rational, determinant decision that is concerned with the essentially inner, subjective states of the victim and the wrongdoer. The proper change in perpetrators’ attitudes, as expressed in, for instance, acts of confession, apology or repentance, on this view, is followed by the victims’ foregoing of resentment and a grant of forgiveness.

In this way, however, the difficulty of forgiveness as a response to the tragic nature of political action is addressed through the traditional philosophical notion of rule, where the appropriate standards for forgiveness are derived from one’s attitudes toward and interaction with one’s self and then imposed upon others and the world from the outside and above (see Arendt 1958, 237–8). In transitional justice and reconciliation debates, accordingly, forgiveness is usually conceptualized with regard to the preconceived end of reconciliation and/or justice. The sovereign penchant can be seen for instance in the broadly liberal insistence on making forgiveness conditioned upon a framework of moral principles which are supposed to mark the advent of a new, just future. Similarly, it characterizes the communitarian bent to conceive of forgiveness as a step that is necessary for the greater good of a harmonious community (see Schaap 2005, 73–5). Yet, confronting the world with a prefabricated, supposedly universal framework of moral standards, this model of forgiveness risks abstracting from its purported goal of understanding the situational factors conditioning a particular action and so missing out on a sustained reflection of how past wrongdoing arose from human embeddedness into a broader field of forces beyond the immediate control of the agent. By implication, it can be said to strangely obscure precisely the potential moral and political value of forgiveness most praised amongst its proponents – its capacity, as an alternative to vindictive resentment and hatred, to realize the possibility of a new beginning, to re-establish relationships between victims and perpetrators and, while acknowledging the seriousness of the wrongs committed, restore in them the sense of responsibility for the shared world.

To substantiate these claims, it merits considering the existentialists’ insights into the political dangers of confronting political affairs with a set of supposedly universal moral standards. The reduction of political judgement into the role of mere determinant application of prefabricated yardsticks onto the particularities of the political world from the outside and above is troubling because it cannot but fail to account for the phenomenal, situated nature of political affairs and threatens to obscure the existence of the public realm. In other words, it risks obviating the process of what Arendt calls “reconciliation with reality,” of understanding and making sense of the world, which corresponds to a fundamental, “existential,” human need and the underlying aim of political judgement (see Arendt 2006, 257; 1994, 307–8). The world of political affairs is grounded upon the constitutive existential condition of human plurality and consists of individuals manifesting their distinct human capacities for action and speech, beginning anew and appearing to each other (Arendt 1958, 55–7). To address the ensuing plurality, contingency and unpredictability of political affairs, Arendt was convinced, abstract standards of thought proved profoundly inadequate. For in attempting to explain and construe the realm of “mere” appearances in terms of supposedly deeper and truer realities, thought to lie above or beneath them, grounding or causing them, they are bound to grow less and less informed by and increasingly distant from particular occurrences and facts in the realm of human affairs and ensue in an atrophied sense of worldly reality. This is politically highly dangerous because humans are essentially worldly beings. As Arendt (1978, 20) writes, humans are not only *in* the world as perceiving subjects, but also *of* the world, as appearances to be perceived by others. As such, they depend on constantly coming to terms with whatever happens and inscribing themselves in meaningful pasts, on kindling a shared sense of the world for the very sense of their own selves as autonomous agents, able to engage with and respond to ever-changing political reality (Arendt 1994, 310; Arendt 2004, 614).

This need is particularly pressing in those moments of transition, of rupture or break in established ways of being in the world brought forth by violence, evil and suffering that can no longer be bridged through appeals to prefabricated frameworks of judgement, but require the whole of society to thoroughly rethink the bases of its identity, its myths, memories and relevant histories (see Ricoeur in Kearney 1995, 37–8). The predominant moral discourse on forgiveness and its reliance on a prefabricated register of absolute standards, in this light, is politically troubling because it short-circuits the situated process of coming to terms with and assuming responsibility for past wrongs. It not only fails to adequately account for the destruction of the common world and the established modes of human interaction brought about by grave wrongs (see Carse and Tirrell 2010, 43–5). Presupposing the existence of a shared moral order between the victims, the perpetrators and the broader society that must first of all be reconstituted through processes of public narration and judgement, it also clouds the political significance of a decision to grant forgiveness, obscuring the crucial questions of in which case and why it might be an appropriate way of reckoning with the past as well as how it would contribute to the building of a just society in the future. Standing at a remove from the public realm, this discourse of forgiveness might indeed seem to amount to an “*a priori* exclusion of criminality and, thus, responsibility” – as Soyinka argues with reference to the South African grant of amnesty (see Soyinka 1999, 31). Worse still, it could be argued in a Nietzschean flair that, thus construed, the discourse of forgiveness threatens to itself assume a somewhat vengeful and domineering air. Through self-centred practices of contrition, apology and repentance it might perversely keep both victims and perpetrators mired by a traumatic past, seeking to escape the ambiguity of worldly struggles into a narcissistic dream of self-righteousness and self-sufficiency, and wary of, or even resentful about, a new future (see Garrard and McNaughton 2010, 26–8).

Against this background, I now turn to delve into the existential aesthetic judging sensibility oriented by the principle of love of the world, highlighting its political significance in its ability to inspire and retain attention on the ambiguous process of reconciling with the burden of a tragic past. Predicated upon a deep-seated awareness of the irreversible loss of eternal standards and absolutes in modernity, the existential aesthetic, worldly sensibility is expressly political in that it, rather than seeking to flee, assumes and faces up to the absurd and ambiguous condition of political judgement as it arises out of the fundamental worldliness or historicity of human political existence. The awareness of the absurd, as developed in the thought of Camus, on the one hand means that we are always already being in the world, that our judgement is situated, enmeshed in a web of worldly relationships – and that it therefore cannot clamour for absolute certainty and transparency, and hope to endow the world with absolute foundations (Camus 1991, 8). On the other hand, however, Camus’s absurd sensibility opens the way for reimagining political judgement as an aesthetic practice, orienting the focus on how to kindle the human judging capacity to engage and endow with meaning the world in its phenomenal particularity and plurality – all the while aware that all of its meanings and values are human and therefore provisional in character (Camus 1991, 89; see also Hayden 2013, 198–9). Aesthetic judgement, in this respect, carries a paramount political significance in that it corresponds to the ability of reflective judgement. As explicitly noted by Arendt (1973, 9), it does not rely on a prefabricated register of abstract standards under which particular facts could simply be subsumed, but calls upon us to “meet the phenomena, so to speak, head-on, without any preconceived system.”

The existential aesthetic judging sensibility thus is well-suited to confront the absurd condition of political judgement after world-shattering suffering and wrongdoing. This is because it answers to the temporal, worldly condition of human existence and is able to cope with the reality of the gap between past and future that, especially in the wake of tragedy and evil, can no longer be bridged by drawing on established standards of thought (see Arendt 2006, 13). For liberated from the quest for deeper causes and realities, purposes and ends, aesthetic judgement affirms human freedom to look upon the past anew and endow with (a general) meaning the particularity of the world of appearances. As Arendt writes, aesthetic judgement is determined neither by “the life interests of the individual nor the moral interests of the self,” but parallels a “disinterested” pleasure or delight at the sight of “the world in its appearance and in its worldliness” (Arendt 2006, 219). In other words, it corresponds to a shift of focus from a concern with the self to an attitude of “loving care” for the things of this world that have no pre-given end, but whose essence is the appearance of freedom in the midst of the world (Arendt 2006, 208, 222; Arendt 1989, 30–1, 76–7). Indeed, the political significance of aesthetic sensibility, according to Arendt, can be traced to its unique capacity of revealing human freedom as a source of worldly events and fostering the view of human beings as actors and sufferers, not passive objects of metaphysical or historical causes or ends (Arendt 1958, 186; Hill 1979, 298). For in this way, aesthetic judgement is able to reclaim the particularity and plurality of past experiences from under the grip of traditional categories and larger wholes, weave them into a meaningful story, and make them part of the common world – thereby establishing the distinctively human significance of politics and also appealing to our capacity for freedom and political action in the future (see Benhabib 1990; Kristeva 2001).

This guiding concern is most explicitly developed in Arendt’s account of aesthetic judgement as “representative thinking” (see Arendt 1989, 43; Arendt 2006, 217, 237). Representative thinking represents a reflective process of moving from the particular to the general that remains always in close contact with the world by tying into its exercise a consideration of a plurality of other perspectives on shared reality. Travelling freely about the world and imagining what it looks like from a plurality of diverse standpoints, representative thinking is able to face up to the ambiguity of political affairs because it gives rise to a perspective of worldly impartiality or of judging “for the world’s sake” (Arendt 2006, 51; Arendt 1968, 7–8). In this way, it can answer to Arendt’s (2006, 219) crucial insight that political judgement concerns “not knowledge or truth,” but “the judicious exchange of opinion about the sphere of public life and the common world, and the decision what manner of action is to be taken in it, as well as how it is to look henceforth, what kind of things are to appear in it” (see also Hayden 2014, 178–9; 2016, 35–7). For remaining loyal to human plurality, representative thinking does not ascend to a “definite,” absolute perspective lying above the realm of political affairs, but judges any particular occurrence “in terms of its position in the world at any given time” (Arendt 1968, 8). In other words, it refrains from the rational, moral quest for completeness and finality to instead retain attention on judgement’s proper aim – that is, on understanding how and why past actions and events came about and echoed in the midst of the common world, thereby engaging in reinvigoration of our sense of worldly reality and thus also confronting the perplexity of responding to them in political action (Arendt 2006, 257; see also Zerilli 2005, 161–3; Buckler 2011, 12, 45–6, 57–8, 107; Fine 2008, 169–70).

In this respect, the narrative, representative judging sensibility can be counterposed to what Camus and Arendt characterize as an attitude of *ressentiment* against the world of political affairs and human condition as such. It is an attitude that came into its own when, after the breakdown of eternal ideas, humans refused wonder at the plurality, contingency and unpredictability of the appearing world and instead sought refuge within their own selves. Consigning the human capacities of judgement and action to the rule of reason that henceforth considered itself absolute, “the human” aspired to transform heaven and earth in accordance with its own blueprint, only to yield to unbridled will to power and nihilistic politics. On this account, the desire to ultimately tame and resolve the ambiguity and contradiction of the political world then also signifies a dangerous disregard for its plural and unpredictable, that is, human character. Worldly judgement, in contrast, is predicated upon an awareness that the independent, complex, at times alien and incomprehensible, nature of the realm of political affairs can never be completely resolved into thought if it is to retain its human character. Accordingly, it commits to ceaselessly reconciling with and creatively responding to its imperfect and tragic nature by illuminating it tentatively and never conclusively, patiently holding fast to the plurality of perspectives on the world and constantly exploring and encountering its boundaries (see Arendt 1994, 183–4; Camus 1970, 148–53, 168–71; see also Zaretsky 2013, 193). The paramount political significance of narrative-inspired worldly judgement, that is, can be said to lie in its ability to reveal on the ruins of history ever anew the contours of a shared, human world, and thereby disclose the possibilities and limitations of political action as they reside in the framework of the public realm and the plurality of perspectives constituting it.

In the third section, I will show how thus construed, narrative-inspired, representative judgement and its underlying purpose of reconciliation with reality can illuminate forgiveness in its political significance by foregrounding it as a situated, worldly practice that is undertaken for the sake of human plurality and the common world. First, however, I explore the suggested shift of focus from a moral to a worldly perspective through two chosen literary works. Thence, I seek to disclose on concrete examples the ways in which the moral, self-centred discourse on forgiveness risks obviating the process of assuming responsibility for the world of human affairs – and how, in turn, the existential aesthetic, worldly judging sensibility’s attentiveness to the situated process of understanding and reconciling with past wrongs ties the decision on the appropriateness of forgiveness to a sustained reflection on how the common world can be rebuilt and how the tragedies of the past can be resisted in the future.

## *The political significance of forgiveness in Pumla Gobodo-Madikizela*’*s* a Human Being Died that Night *and Vitomil Zupan*’*s* Minuet for Guitar

*A Human Being Died That Night* is Gobodo-Madikizela’s account of her visits to and conversations with Eugene de Kock, considered to be one of the most notorious executors of the brutality of Apartheid in South Africa, a reputation that earned him the nickname of “Prime Evil.” The commanding officer of the counter-insurgency police unit, responsible for countless killings, torture and murder of resistance activists, de Kock was later tried and sentenced to a life in prison. Gobodo-Madikizela’s (2004, 52–7) journey in understanding focuses on “Apartheid’s grand plan of corruption,” trying to see how it might have conditioned de Kock’s actions and suppressed his conscience and knowledge of right and wrong. She acknowledges that de Kock exercised free will in committing the atrocities and should therefore be condemned, yet also draws attention to “the sophistication and subtlety with which apartheid drew its followers to support its mission” (Gobodo-Madikizela 2004, 59). She acknowledges how the system worked to rationalize murder as a necessary step in a legitimate fight against the enemy, while at the same time blurring the lines of accountability, “creating a grey zone of deniability that made it exceedingly difficult to determine the chain of command,” and allowing political leaders to deny any responsibility for past crimes (as well as the broader systemic forces “hatching” them) (Gobodo-Madikizela 2004, 65). De Kock himself notes how even though “inherently you know that killing is not right,” it was also “sanctioned by the highest authority” (Gobodo-Madikizela 2004, 74–5). He relates how during the killings he would shut off his awareness of the human faces and his sense of reality, embalming them within the air of “the surreal” (Gobodo-Madikizela 2004, 76–7). His account also expresses bitterness against the politicians, leaving him alone with a sense of disenchantment at having “fought for nothing” and the terrible ordeal to confront his past (Gobodo-Madikizela 2004, 41, 78).

Considering the way de Kock’s conscience was shaped and suppressed by his situation, Gobodo-Madikizela (2004, 58–9) concludes that despite the horrific nature of their crimes, some perpetrators “may need our sympathy, because under corrupt leadership they lacked appropriate models to stir them away from a violent path.” De Kock, too, is clearly repentant, suffering under the unbearable weight of the past, expressing “genuine remorse and regret over destroying lives and severing the relationships that were connected to them” and carrying “the voice of an outcast begging to rejoin the world of the living” (see Gobodo-Madikizela 2004, 114–16, 121). Touching his hand and feeling pity for him as “a human being capable of feeling, crying and knowing pain,” however, also leaves the author ill at ease (Gobodo-Madikizela 2004, 35–8). The project of identifying with the perpetrator, of being drawn into his world, which brings her into direct contact with the possibility of evil within herself and further blurs any clear-cut boundaries between right and wrong, ensues in the feelings of discomfort and fear (Gobodo-Madikizela 2004, 35–8, 116, 123).

The willingness to engage with and speak to the perpetrator, in Gobodo-Madikizela’s account, then opens the possibility of setting his actions in a broader political context, revealing how they were conditioned and even governed by external forces beyond the control of the agent, and thereby also of seeing behind the monstrous deeds once again a human being (see Gobodo-Madikizela 2004, 119–20). Yet, her focus also remains directed on unveiling the inner “moral sensibility” of the perpetrator, before concealed “under the façade of ‘obedience to orders’ or righteous ‘duty to my country’,” and on encouraging him to recognize “what he has done,” and also that “all along, he knew that he was human and knew right from wrong” (Gobodo-Madikizela 2004, 119–20, 123). Gobodo-Madikizela, to be sure, shies away from prescribing forgiveness, and instead links the forgiving disposition and willingness to engage in dialogue to the challenge of creating conditions for the practice of building new relationships and restoring broken ones – a challenge, she notes, not properly appreciated and addressed within the orientation of retributive justice, narrowly focused on a restoration of political and legal order and on “making sure that people remain or are put where they belong” (see Gobodo-Madikizela 2004, 97, 126–7). Nevertheless, she ties the judgement on whether or not to forgive to a change in psychological states (Gobodo-Madikizela 2004, 95–7). Just as forgiveness would seem to be conditioned upon remorse on the part of the perpetrators, its being granted is linked to an expression of sympathy on the part of the victims (Gobodo-Madikizela 2004, 124–5, 98, 137–9). By expressing remorse, it is held, the perpetrators come to acknowledge in the previously unseen or disregarded perspectives of the victims human beings whose humanity was unjustly denied (Gobodo-Madikizela 2004, 128, 130). Victims, in turn, are inspired to empathetic understanding, granting forgiveness as a compassionate response to another human being in pain and thereby restoring the offender back among the ranks of the human community – even when a normative framework of moral principles of justice would seem to preclude this possibility (Gobodo-Madikizela 2004, 126–9). Thus the victims and the perpetrators are mutually affirmed in their common humanity. This newly restored coincidence of human consciousnesses that “seal[s] the cracks” caused by past wrongs replaces the cycles of violence and revenge with a vocabulary of tolerance, friendship and dialogue and leads to societal transformation and reconciliation (Gobodo-Madikizela 2004, 129, 126, 132–3).

Relying on a “moral compass” already in place and imagining the practice of forgiveness as a play between pure consciousnesses removed from their worldly existence, however, this account in fact begs the question of the political significance of forgiveness. Quite apart from the difficulty of verifying the authenticity of expressions of remorse, we forgive simply because of the kind of compassionate and empathetic people that we are and because of, for instance, our preference for or commitment to values of solidarity, love and good will (see also Garrard and McNaughton 2010, 116–21; Griswold 2007, 65–6). What is thus obviated is precisely the challenge that the often unimaginable instances of wrongdoing pose to our faculty of judging on the appropriateness of forgiveness as well as the implications of this decision for the political world and our ways of interacting with each other. Far from encouraging a renewal of relationship with the world and others, this take on forgiveness in fact leaves the perpetrator suffering under the weight of the past, alone and resentful of the future, and risks placing the victim/spectator into a posture of self-righteousness and moral superiority that shies away from coming to terms with the tragic character of political action. Behind the banner of a perhaps unwitting presupposition of an easy closure, it reveals a continued refusal to assume responsibility for, and engage in shared struggles in, the world of human affairs.

In this respect, Gobodo-Madikizela’s account can be helpfully contrasted with Vitomil Zupan’s novel, *Minuet for Guitar.* The novel tells the story of the partisan soldier Berk and his experience of the horrid and senseless reality of war as a member of the guerrilla resistance army during the Second-World-War Axis occupation of Yugoslavia. Years later, during a holiday in Spain, Berk encounters Joseph Bitter, an ex-German soldier and his former enemy. Parallel to and in many ways in conversation with Berk’s recounting of the horrors, absurdities and ironies of war, runs his attempt to engage Bitter in a dialogic, shared endeavour to make sense of the past. While avoiding any explicit acts of apology and forgiveness, the novel arguably powerfully discloses a relational, worldly judging orientation as well as the striving to come to terms with the tragic character of the world it embodies.

In contrast to Gobodo-Madikizela’s account, Berk’s memories of war are first and foremost characterized by a disconcerting and anxiety-inducing flight of meaning. Through a scattered and purposeless depiction of endless marches and ambushes, hunger and exhaustion, cruelty and killings, interspersed with introspective reflections, surreal images and allusions to the broader historical context, the novel all too faithfully reflects the author’s perplexing position as a consciously free and responsible human being suddenly caught in the midst of foreign and hostile forces beyond his control and calls into question any settled perspective or standard of judgement from which to gain a comprehensible view of events and distinguish good from evil (Zupan 2011, 53, 112–13). Crucially accompanying Berk’s absurd sensibility, however, is his deeply entrenched commitment, felt throughout the novel, to human solidarity, his need to weave bonds of fellowship as the only remaining route to a meaningful human life. Once he encounters Bitter, sensing he could have been a soldier during the Second World War, he is drawn to him, it seems, out of an existential, not fully articulate, yet pressing need to converse with him, to relive the memories of the war with him and thereby reach a better understanding of its inexplicable and senseless character (Zupan 2011, 34–5, 81). Thus Berk: “I did not yet know his name was Bitter. He walked and I walked after him, as if we belonged together” (Zupan 2011, 34).

Lying about his nationality and his own part in the war, Berk slowly learns that Bitter was indeed a German Army soldier, for a time also fighting in the territory of (ex-)Yugoslavia. At first highly reticent about himself, Bitter too soon reveals “a growing pleasure in talking about the war” and finds in Berk a much needed companion (Zupan 2011, 146, 155–6). In his reflections, however, he views the war purely from a military perspective. It is the standard of success that guides his rumination on military tactics, strategy, and even on the human elements of fear and terror and a sense of comradeship among the soldiers (Zupan 2011, 154–8). To Berk’s question of “[h]ow was it that the Germans decided to occupy so much foreign territory,” his response is: “well, wasn’t it the same with any army in history?” (Zupan 2011, 220). And further: “blood is spilled in all wars, atrocities occur, people die, settlements are destroyed, wide-eyed children meet a new way of life [...] there is nothing new in this” (Zupan 2011, 148). When the question of guerrilla (resistance) fighters is brought up, Bitter refers to them as “insects” and “bandits,” whose “improvisation” in fighting and “irresponsible” forms of attack made them “a hard nut for a regular army to crack” (Zupan 2011, 124–5, 146, 120). He continues, “[o]f course, it was the hostages that paid for it” (Zupan 2011, 147). And even: “mopping up would have been easier if they had not had the civilian population on their side. You can’t exterminate them all, you know. Then of course if we’d had more time” (Zupan 2011, 125). Within his military frame of mind, in short, Bitter seems unable to even pose the question of whether and how the occupation and his participation in it might have been wrong, avoiding any moral and political judgement on the legitimacy of means employed or ends pursued.

Berk, for his part, shies away from casting Bitter into the role of an evil occupier and regards him as a human being similarly engulfed and subjected to the inhumanity of larger forces of history. He acknowledges, for instance, that Bitter, too, had suffered, “that the Allies had bombed him out of house and home,” or that he still endures the consequences of an injury caused by a guerrilla bomb attack: “Both of us, my dear Bitter, were mere pawns in the war” (Zupan 2011, 37, 146–7, 148). Further, he self-reflectively ponders about the forms of political mentality ruling Yugoslav politics where the commitment to highest ideals hid a low-life opportunism and chauvinism – setting up the conditions for foreign occupation to also lead to a bloody internal war (Zupan 2011, 131, 274–5; 278–9, 54–5). Similarly, he admits that he too regarded the feelings of hatred at the enemy a necessary part of the resistance struggle and was unable to discern a human face behind the green helmets. Nevertheless, behind his conscious refusal to divide the world and people into categories of good and evil, there remains, it seems, a growing, even though not clearly expressed, tinge of accusation at, even contempt for, his former enemy. What he refuses to accept, mocks and indirectly condemns is Bitter’s lack of reflexivity and his thoughtlessness, his inability (or unwillingness) to acknowledge or even inquire into how it was precisely the blind obedience to superior orders and an uncritical loyalty to higher abstract principles of, for instance, justice, god, nation or führer, that was able to lead humanity into an unprecedented scourge of hatred, torture and murder (see Zupan 2011, e.g. 237, 147, 274–5, 54–5; 278–9). What he wishes to make Bitter realize, in other words, is the effects his seemingly benign adherence to authority produced in the world and among other human beings.

The conversation then in a way leads to a reinstatement of the old front between enemies, at no point more clearly evident than when Bitter finds that Berk is actually a Yugoslav (see Zupan 2011, 311–12, 323, 338–9). Yet, this realization also ensues in a certain reframing of old hostilities and in at least a limited step towards mutual recognition. Bitter goes first: “‘I suppose you were in the Partisans?’ ‘*Bei den Banditen?*’ ‘*Bei den Partisanen*, *hab*’ *ich g*’*sagt*’*.*” And Berk similarly: “‘I suppose you were a member of the German National Socialist Party?’ ‘*Ein Mitglied der blutigen Hitler-Partei?*’ *Ein Mitgleid der N.S.D.A.P*, *hab*’ *ich g*’*sagt*’” (to which Bitter replies in the negative) (Zupan 2011, 338–9). It is this step that can be said to enable them to explicitly address the question of the meaning of past events for their lives in the present and for the future. Later on, Berk finds out that, after the war, Bitter was suffering from pangs of guilt and struggling to understand how and why “can it happen that a perfectly normal, orderly human being becomes the exact opposite” (Zupan 2011, 365). Nevertheless, as a German citizen and soldier he refuses to see any linkage between his actions and the ascending spiral of Nazi measures and politics that led to war (and Holocaust). Escaping into the tragic embrace of collective guilt, while rejecting any personal accountability, he assumes that the issue of political judgement and of understanding was satisfactorily dealt with with the punishment of war criminals at Nuremberg Trials, imposing no further imperatives on how to avoid such catastrophes in the future (see Zupan 2011, 339). Berk, on the contrary, refuses to reduce the unprecedented “edict of cruelty, a plan for ruthlessness and atrocity, an order that enemies should perish in night and fog, without trace” that characterized the past, to an issue of eternal and seemingly necessary character of war or to human animality (Zupan 2011, 342, 366–7). He accordingly states: “So it’s not only a matter of war as such, Herr Bitter. And it is also true that not all wars are the same” (Zupan 2011, 342). Parallel to his efforts at understanding the past, thus, runs his pronounced awareness of the need to remain attentive to and respond to the exigencies and dangers of the present political situation, above all the lingering “ideological blizzard that besets the world” (Zupan 2011, 341). To these ruminations, Bitter replies simply: “You’re an unhappy man, aren’t you?” (Zupan 2011, 341). Berk’s continued refusal “to fraternize with the enemy” (see Zupan 2011, 367), then, seems more likely a response to Bitter’s complacency in the present, his refusal to engage his freedom in the world, to recognize what happened and assume responsibility for it, rather than any of the specific crimes he committed in the past.

While affirming his separateness from Bitter, their shared ruminations on the war nevertheless lead Berk to sharpen his insights into how, in the midst of the often overwhelming meaninglessness, atrocity, political crime, stupidity and shallowness ruling his time, it might still be possible to rebuild and sustain a human world. In particular, he is insistent on upholding the value and keeping alive the memory of ties of comradeship and “family warmth” woven among the partisan resistance fighters involved in what he affirms to be a legitimate fight against the foreign occupiers (Zupan 2011, 317, 166–7, 279, 269). Especially important in this respect is his friendship with Anton, with whom they form “a new, common self,” “like two drops of water which come close to each other and then, after a momentary tremor, leap together to form one single drop” (Zupan 2011, 329, 291). It is a unity born of and sustained through “agreement” as well as “clashing and rebounding,” “an atmosphere of possessiveness and liberation” – where neither of the two was any longer a self-sustaining, separate self, but depended on the other for understanding and meaning, yet which nevertheless was not reducible to the pursuit of a higher end that would eliminate their individuality and difference (see Zupan 2011, 329, 291–2, 279, 297–9). Berk’s emphasis on the forms of human solidarity, on the one hand, thus makes him particularly alert and wary against the quickly shaping tendency within the liberation movement to render the goal of freedom into a new ideological end of political action to which all individuality must be sacrificed and for the achievement of which all means are justified (see Zupan 2011, 357–62). On the other hand, however, the experience of human solidarity also affirms Berk in the awareness that a proper response to the absurd “degeneration of our ideals,” does not lie in an embittered and contemptuous individualistic escape from new wars, suspicion, double-talk, and cunning brewing on the horizon. Instead, it rests in a continuous and often painful effort to create islands of humanity in the midst of dehumanizing forces of history by a steadfast refusal to forget and a willingness to share and exchange one’s experiences with others (Zupan 2011, e.g. 369, 383–7). Berk’s realization of both the utmost importance and difficulty of engaging and speaking with his former enemy, gains a parallel confirmation in his somber, yet vigilant promise that, shorn of long lost ideals, refuses to give up on the world of human affairs: “See you in the next war.”

## The Political Significance of Forgiveness, Love of the World, and the Ambiguity of Assuming Responsibility for Past Wrongs

The paramount importance of the representative judging capacity for thinking the political significance of forgiveness then can be said to lie in its willingness to depart from any pre-established standard, including the lures of empathy, and open itself to consider the outside world and separate others in their particularity. For in its acts of imaginative world-travelling, of actively reclaiming a plurality of memories on the past, worldly judgement first of all allows for “things [to] *become* public,” for painful pasts to become a part of shared reality (see Zerilli 2012, 21–2, 23) – and is thus already engaged in the tentative process of reconciling with reality. As such, it is especially relevant to face up to the challenge of forgiveness in instances of grave suffering and wrongdoing that – as alluded to above – do not simply represent a violation of a pre-given and valued moral principle, but a thorough shattering of the very world, of shared modes of interaction and meaning, within which the crimes and experiences of loss and suffering could be recognized and vindicated (Carse and Tirrell 2010, 51–2). As Carse and Tirrell (2010, 49) argue drawing on the statements of the survivors of the Rwandan genocide, the sense of disorientation, shattered identity and chaos after grave wrongs corresponds to being “betrayed by life.” Within this horizon, the rational, self-centred model of forgiveness is inadequate: its reliance on deliberate gestures of apology and repentance, that is, can find no secure ground of the world that would bestow on these acts an intersubjectively shared significance and foreground them as meaningful responses to a painful past (Carse and Tirrell 2010, 45). Carse and Tirrell (2010, 46–7) note an example of a survivor of the Rwandan genocide who chose to forgive so as to “find tranquillity” and not to “suffer my whole life long asking myself why they tried to cut me.” Yet, this example of forgiveness mirrors a “unilateral shift,” explicitly directed at survivor’s inner peace and healing that at the same time manifests an abandonment of “all hope in the possibility of reconciliation and mutual understanding” (Carse and Tirrell 2010, 47). Many other testimonies, in contrast, affirm the meaninglessness and offensiveness of forgiveness in light of the outrageous crimes: “I can’t imagine any forgiveness capable of drying up all this spilled blood” (Carse and Tirrell 2010, 50, 44; see also Staub and Pearlman 2001, 207).

On this basis and similarly to the existential account of worldly judgement, Carse and Tirrell (2010) call for a shift to a model of forgiveness that is based on practices of “world-building.” This view emphasizes the importance of “generating” a shared framework or ground within which the victims and perpetrators can move from radical distrust and apathy to the development of relationships of mutual recognition and understanding, and also reclaim their capacity of active engagement with and in the world (Carse and Tirrell 2010, 50–1). As such, it is “inherently relational” in its insistence that the possibility of moral repair only lies “in relationship with, rather than disconnection from,” others (Carse and Tirrell 2010, 48, 50). The existential worldly judging sensibility is well-suited to confront this relational character of forgiveness because, as Arendt (1989, 43; 2006, 51) explicitly points out, it does not involve a desire to penetrate the ultimate and innermost subjectivity of the perpetrators and simply adopt their views – a tendency, as we have seen, that can be morally compromising or damaging for the victims and the broader circle of spectators, and leave all sides alienated from the common world (Carse and Tirrell 2010, 58)**.** Worldly judgement’s engagement with the perpetrators instead is intent on viewing the world from their perspective, considering the possibilities (and alternative courses of action) available to them as well as how they themselves made sense of their lives, choices and crimes (see Schaap 2005, 70, 72; LaCaze 2014, 214; Griswold 2007, 87–8). By the same token, as well demonstrated in Berk’s dialogue with Bitter, representative judgement on whether or not to forgive does not amount to a complacent and self-righteous moralism that would exhaust itself in a moral denunciation of a painful past. Instead, it implies a willingness to undertake a critical self-scrutiny of one’s own actions and the broader field of relationships and structures that conditioned them. As such, it helps to displace the crude victim/perpetrator dichotomy that usually lingers in the wake of mass injustice and conflict, showing how many conflicts may not allow for any clear-cut divisions between right and wrong (Scott 2010, 12–13). For while the partners in dialogue on this view may well remain on opposite poles, the representative judging sensibility nevertheless discloses a common world laying in-between them, prying open a space for shared reflection on how worldly reality had similarly placed them all at the mercy of alien, even incomprehensible forces beyond their control.

Remaining loyal to the plurality of the political world, then, representative judgement displaces the focus on penetrating the ultimate nature of the experience of suffering and injustice and reaching an easy, conclusive attribution of blame. Rather, it retains attention on disclosing particular actions and events in their worldly appearance, letting their meaning (or value judgement) surface tentatively, and never unambiguously, out of a consideration of both how they emerged “in the midst of human society,” within the web of human relationships, and of how they echoed in the common world, how they bore upon the human, political status of a plurality of individuals constituting it (see Arendt 1994, 404). In this way, representative, worldly judgement also reveals the distinctly political significance of a decision on whether or not to forgive by foregrounding the need for it to be arrived at dialogically and from within the worldly situation, rather than pronounced from on high. For in confronting past wrongdoings and sufferings in their appearance in the world, on the one hand, it allows for an appreciation of the tragedy of political action, disclosing the grounds for forgiveness in how, conditioned and perhaps suffused by the broader field of worldly relationships, human engagement in the world is able to produce a plethora of unforeseen consequences. It can, for example, trace the ways in which individuals’ embeddedness in the world, in particular in the broader field of unjust and oppressive relationships or institutions, may have made their perhaps seemingly benign actions ensue in radical denials of freedom and humanity of certain individuals or groups. On the other hand, it is also attuned to a careful drawing of distinctions as to the wrongfulness of the past crimes. It is able to differentiate, for instance, between those offences that lie “in the very nature of action’s constant establishment of new relationships within a web of relations,” and those that are destructive of the political realm and constitute an affront against the human status (Arendt 1958, 240; see also LaCaze 2014, 213).

In revealing the source of perhaps previously unimaginable suffering and unprecedented wrongdoing in the tragedy of human involvement in the world, worldly judgement thus also refrains from envisioning forgiveness in terms of a pre-determined, moral end. Instead, it foregrounds it as a continuous, interactive process which, prior to (or even more importantly than) explicit acts of repentance and forgiveness, involves gestures and practices oriented towards sharing, understanding and rebuilding of the common world (see also Griswold 2007, 98; Carse and Tirrell 2010, 55–6; Staub and Pearlman 2001, 215–16, 221).^4^ As such, however, the process of forgiveness also is marred by complexity, inevitably bearing the elements of risk and uncertainty (Staub and Pearlman 2001, 218; Carse and Tirrell 2010, 58). The existential aesthetic sensibility explicitly brings out and tackles this political, ambiguous dimension, by foregrounding forgiveness as a practice undertaken for the sake of human plurality and of the common world**.** Focusing on the worldly environment of past wrongdoing and suffering, it refuses to fixate perpetrators in the role of either inherently monstrous villains or helpless victims of inhuman forces. Similarly, representative judgement on whether or not to forgive resists the prevalent moral tendency to tie the affirmation of the victims’ identity and dignity to a pre-fabricated prescription on what former wrongdoers are supposed to be like or how they are supposed to change. Instead, it ties former enemies indissolubly together through the mediation of the shared reality, fostering the possibility for their recognizing each other as equal members of the common world and capable of affirming their freedom in the future. Eschewing the quest for a complete coincidence or final communion between human consciousnesses (e.g. Camus 1971, 130), that is, the narrative judging sensibility contains an appeal to the freedom of both victims and the perpetrators to engage itself in the world and participate in the shared endeavour to rebuild relationships and institutions in ways that broaden and foster, rather than restrain, the space for mutual recognition and the human ability to be free together. This worldly orientation is particularly significant in those frequent cases where two or more sides are engulfed in an ever-widening spiral of mutual denunciations, hatred and violence (Staub and Pearlman 2001, 206, 217). For furthering an appreciation of how the crimes of one side fuel and even offer justification for the crimes of the other, it may inspire all parties to the conflict to engage in forms of action that would appease, rather than provoke, further violence. At the same time, the activity of judging for the sake of the world also remains attentive to the possibility of individuals (or groups) whose actions have (or continue to) so radically obliterated human plurality and the shared reality that they cannot be forgiven and reconciled with as equal members of the common world.

The existential aesthetic judgement oriented by the principle of love of the world, to be sure, avoids issuing a technical prescription or a clear-cut guideline on whether and when forgiveness should be awarded, affirming the ambiguity of a new beginning. Yet, in its attention to the situated process of reconciling with past wrongs, it is able to bring out forgiveness in its paramount political significance, showing how it contains the possibility of re-establishing relationships with others and the common world (see Arendt 1958, 237, 241). For what it displaces is the assumption still permeating the predominant moral discourse on forgiveness, that one’s intentions can be neatly separated from the broader field of worldly processes and interactions conditioning an action and causing for it to produce a plethora of unforeseen consequences. This presumption is problematic because, while recognizing the worldly context of past wrongs, it also acknowledges the fact of our embeddedness in a web of forces beyond our control only insofar as it is said to release us from the burden of assuming responsibility for our actions, that is, to a certain extent, let us “off the hook” (see Card 1996, 22–3). As such, it contains a variation of the traditional philosophical claim to autonomy. This claim, for instance, can be seen in attempts to reduce the burden of assuming responsibility for the past to the matter of ascribing judicial culpability and holding the perpetrators absolutely accountable for their actions. Yet, the challenge of coming to terms with painful pasts might similarly be obscured if the process of assuming responsibility is consigned to an essentially inner reckoning with one’s conscience and envisioned to proceed on the basis of public expressions of remorse, repentance and atonement as manifestations of the required (and predetermined) change in the moral identity of the wrongdoers (and bystanders) (see Schaap 2001, 757). For what is again missed is precisely what is involved in the situated, ambiguous process of reconciling with the tragic nature of political action: how to affirm the reality of human freedom in the midst of an untameable world and assume responsibility for events and occurrences that we had never wished or intended, yet that nevertheless and irreversibly constitute a part of our shared reality (see Zerilli 2005, 163; Herzog 2014, 186; Kruks 2012, 34–5).

Worldly judgement, in this respect, resists the temptation to conceive of forgiveness as a sovereign act, by which the human subjectivity is imagined to be able to take upon its shoulders the whole brunt of responsibility for itself and the world, and reach an ultimate redemption for past crimes. For constantly disclosing the world in its particular and plural character, it remains ever attentive to how this temptation may in fact alleviate a sense of responsibility for our shared reality – not only rendering unnecessary the continued efforts to engage in and with the world and respond to its challenges, but also leading to a willingness to accept further erasures and injustice as a legitimate and necessary path towards final reconciliation (see Hayden 2009, 10–31; Muldoon 2009, 11). Instead, orienting the appropriateness and practice of forgiveness by the principle of “for the sake of the world,” it is able to trace the tragedy of the past to the very conditions ruling the world of political affairs, and thereby disclose how suffering and wrongdoing were made possible precisely by human interdependence and the ensuing vulnerability that represent the most distinct characteristics of our sharing-the-world-with-others (see also Griswold 2007, 49, 110, 133–5). By implication, representative judgement oriented by love of the world – as Card suggests in her discussion of moral luck – directs attention to and kindles our sense of responsibility for the forms of relationships and community nurtured and sustained as well as to the medium of political structures and institutions, and how they work to constrain or widen the possibilities for individual judgement and action (see Card 1996, x, 22–3). While acknowledging suffering as an inevitable part of our embodied, worldly existence, the political significance of forgiveness, in this respect, lies in its ability to constantly rediscover the world as a source of human solidarity as the only point of support in the fight against its tragic nature (Camus 1995, 28) – directing attention to the question of what kinds of relationships and forms of community, that is, the in-betweens of the world, between former enemies can and should be rebuilt, nurtured and sustained.

## Concluding Thoughts: Forgiveness and Coming to Terms with the Tragic Nature of Political Action

The article has sought to explore the political challenge and significance of forgiveness as an indispensable response to the inherently imperfect and tragic nature of human, political life through the lens of the existential narrative-inspired representative judging sensibility. For this purpose, it drew on its ability to kindle the process of coming to terms with the tragedy of political action, of reconciling with the world that, especially in the wake of mass injustice and suffering, dons the appearance of an absurd, impenetrable and shapeless weight, devoid of human significance. The existential worldly judging sensibility, I argued, is particularly well-suited to confront this challenge because it is able to engage reality in its particularity, plurality and unpredictability, disclose how past suffering and wrongdoing arose from the perplexity of human engagement in the world, and thereby also constantly illuminate the political world as a shared, human world. In its world-building capacity, thus, it brought to light the distinctly political significance of forgiveness by foregrounding it as a practice undertaken for the sake of human plurality and the common world. Grounded in the situated, ambiguous process of reconciling with and assuming responsibility for past wrongs, worldly judgement revealed that forgiveness cannot purport to ultimately mend, perfect, contain or flee the imperfect and tragic nature of the world, precluding the possibility of an ultimate redemption for past crimes. Tracing the tragedy of the past to the very conditions ruling the world of political affairs, instead, it honestly confronted the main political challenge of forgiveness in a recognition that the outside world and separate others do not represent a hindrance, but the very condition, for good or bad, of our freedom. While acknowledging suffering as an inevitable part of our embodied, worldly existence, the political significance of forgiveness, in this respect, can be said to lie in a determination to confront the tragedy of politics by appealing to the promise of human solidarity, constantly striving to create the space for dialogue, further mutual understanding, and build relationships and institutions that are welcoming to human plurality.

## Footnotes

1 In this respect, this article corresponds to the recent attempts, in for instance Mihai and Thaler (2014), to examine the distinct nature and challenges of *political* apology. See especially MacLachlan (2014), Mihai (2013), and Thaler (2012).

2 The true horror of totalitarianism, for Arendt, lay not simply in the sheer horrendousness of its crimes, but in the fact that it has definitely “brought to light the ruin of our categories of thought and standards of judgement” (Arendt 1994, 318). For in the wake of its terrifying neglect for all utilitarian considerations, of crimes that could not be traced to mere quest for power for power’s sake, nor to “any other humanly understandable sinfulness,” and of its general “arrogant emancipation from reality and experience,” not only the traditional categories of guilt and innocence, but the very distinction between truth and falsehood, were left curiously devoid of all meaning (Arendt 1994, 350, 354–5). The evil of totalitarianism, for Arendt, instead rested in its attempt to destroy the very humanity in human beings – what she called the making of human beings superfluous *as human beings* (Arendt 2004, 620).

3 This proposition is often manifested in the appeal of “there but for the grace of God go I″ (see e.g. Thomas 2009, 127–31).

4 Examining the possibilities for forgiveness in the aftermath of the Rwandian genocide, Carse and Tirrell (2010, 53–6) and Staub and Pearlman (2001, 221, 224–5), for instance, emphasize the importance of community groups that promote an exchange between the victims, perpetrators and the broader public, either in the form of engaging them in joint projects, or in the sharing of painful experiences and understanding of the situational factors that made the genocide possible**.**
